# Partnered implementation of the veteran sponsorship initiative: protocol for a randomized hybrid type 2 effectiveness—implementation trial

**DOI:** 10.1186/s13012-022-01212-9

**Published:** 2022-07-08

**Authors:** Joseph C. Geraci, Erin P. Finley, Emily R. Edwards, Sheila Frankfurt, A. Solomon Kurz, Nipa Kamdar, Megan E. Vanneman, Leonard M. Lopoo, Hannah Patnaik, Jean Yoon, Nicholas Armstrong, Ashley L. Greene, Gilly Cantor, Joseph Wrobleski, Erin Young, Matthew Goldsmith, Richard W. Seim, Marianne Goodman

**Affiliations:** 1 Transitioning Servicemember/Veteran And Suicide Prevention Center (TASC), VISN 2 Mental Illness Research, Education and Clinical Center, James J. Peters VA Medical Center, Bronx, NY USA; 2 Center of Excellence for Research on Returning War Veterans, VISN 17, Doris Miller VA Medical Center, Waco, TX USA; 3 Resilience Center for Veterans & Families, Teachers College, Columbia University, New York, NY USA; 4 Department of Psychiatry, Icahn School of Medicine at Mount Sinai, New York, NY USA; 5 Center for the Study of Healthcare Innovation, Implementation, and Policy (CSHIIP), VA Greater Los Angeles Healthcare System, New York, USA; 6 Central Texas Veterans Healthcare System, Temple, TX USA; 7 Center for Innovations in Quality, Effectiveness and Safety, VA, VA Houston, USA; 8 Informatics, Decision-Enhancement and Analytic Sciences (IDEAS) Center, VA Salt Lake City Health Care System, Salt Lake City, UT USA; 9 Division of Epidemiology, Department of Internal Medicine, University of Utah School of Medicine, Salt Lake City, UT USA; 10 Division of Health System Innovation and Research, Department of Population Health Sciences, University of Utah School of Medicine, Salt Lake City, UT USA; 11 Department of Public Administration and International Affairs, Syracuse University, Syracuse, NY USA; 12 VA Health Economics Resource Center, VA Palo Alto Healthcare System, Livermore, CA USA; 13 Center for Primary Care and Outcomes Research, Stanford University, Palo Alto, CA USA; 14 Institute for Veterans and Military Families, Syracuse University, Syracuse, NY USA; 15 Global Mental Health Lab, Teachers College, Columbia University, New York, NY USA

**Keywords:** Veteran Sponsorship Initiative, Reintegration difficulties, Suicide prevention, Connectedness, VA utilization, Community intervention, Stepped wedge

## Abstract

**Background:**

The USA is undergoing a suicide epidemic for its youngest Veterans (18-to-34-years-old) as their suicide rate has almost doubled since 2001. Veterans are at the highest risk during their first-year post-discharge, thus creating a “deadly gap.” In response, the nation has developed strategies that emphasize a preventive, universal, and public health approach and embrace the value of community interventions. The three-step theory of suicide suggests that community interventions that reduce reintegration difficulties and promote connectedness for Veterans as they transition to civilian life have the greatest likelihood of reducing suicide. Recent research shows that the effectiveness of community interventions can be enhanced when augmented by volunteer and certified sponsors (1-on-1) who actively engage with Veterans, as part of the Veteran Sponsorship Initiative (VSI).

**Method/design:**

The purpose of this randomized hybrid type 2 effectiveness-implementation trial is to evaluate the implementation of the VSI in six cities in Texas in collaboration with the US Departments of Defense, Labor and Veterans Affairs, Texas government, and local stakeholders. Texas is an optimal location for this large-scale implementation as it has the second largest population of these young Veterans and is home to the largest US military installation, Fort Hood. The first aim is to determine the effectiveness of the VSI, as evidenced by measures of reintegration difficulties, health/psychological distress, VA healthcare utilization, connectedness, and suicidal risk. The second aim is to determine the feasibility and potential utility of a stakeholder-engaged plan for implementing the VSI in Texas with the intent of future expansion in more states. The evaluators will use a stepped wedge design with a sequential roll-out to participating cities over time. Participants (*n*=630) will be enrolled on military installations six months prior to discharge. Implementation efforts will draw upon a bundled implementation strategy that includes strategies such as ongoing training, implementation facilitation, and audit and feedback. Formative and summative evaluations will be guided by the Reach, Effectiveness, Adoption, Implementation, and Maintenance (RE-AIM) framework and will include interviews with participants and periodic reflections with key stakeholders to longitudinally identify barriers and facilitators to implementation.

**Discussion:**

This evaluation will have important implications for the national implementation of community interventions that address the epidemic of Veteran suicide. Aligned with the Evidence Act, it is the first large-scale implementation of an evidence-based practice that conducts a thorough assessment of TSMVs during the “deadly gap.”

**Trial registration:**

ClinicalTrials.gov ID number: NCT05224440. Registered on 04 February 2022.

**Supplementary Information:**

The online version contains supplementary material available at 10.1186/s13012-022-01212-9.

Contributions to the literature

- The USA is undergoing a suicide epidemic for its youngest Veterans (18-to-34-years-old) as their suicide rate has almost doubled since 2001. Veterans are at the highest risk during their first-year post-discharge, thus creating a “deadly gap” for them.
- A cluster randomized, stepped wedge, hybrid type 2 effectiveness-implementation design will evaluate the effectiveness and implementation outcomes of the Veteran Sponsorship Initiative.
- Mixed-method examination of implementation strategies will directly inform national efforts to implement community-based interventions to address the epidemic of Veteran suicide.

## Background

**Fig. 1:**
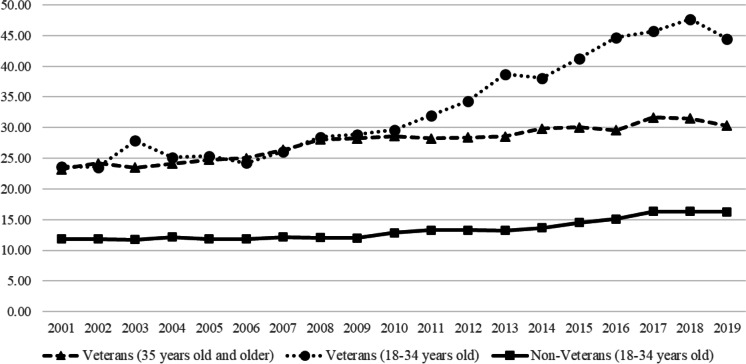
Suicide Rates (per 100,000) Based on Age and Veteran Status [74]

The United States’ youngest Veterans are experiencing a suicide epidemic. Suicide rates for Veterans aged 18 to 34 nearly doubled between 2001 and 2019, from 23.6 per 100,000 to 44.4 per 100,000 [74]. In 2019, these rates were 2.73 times higher than same-aged non-Veterans and 1.65 times higher than older Veterans (see Fig. 1).

These elevations may be due, at least in part, to elevated risk for suicide during the transition from active-duty military service to civilian life. Approximately 200,000 servicemembers exit active-duty military service each year [71]. In a study of military personnel exiting service between 2001 and 2011, suicide rates were nearly three times higher during the first year following military separation compared to active duty and remained elevated up to 6 years following separation [57]. Correspondingly, some research suggests a positive association between severity of reintegration difficulties and risk of suicidal ideation [35]. The period between military discharge and successful reintegration into civilian life is therefore referred to as a “deadly gap” characterized by a relative gap in support and service and corresponding increase in risk for suicide [23, 60].

To date, VA-based suicide prevention initiatives have had limited utility, because only 26% of newly separated servicemembers enroll in VA healthcare [72]. Further, up to 70% of servicemembers and Veterans who die by suicide deny suicidal ideation when assessed by healthcare professionals in the months preceding their death [6, 39].

In response to these challenges, the White House [83] recently encouraged preventative, public health approaches that utilize public-private partnerships between government agencies and community-based organizations to reduce servicemember and Veteran suicide. Accordingly, the VA implemented a public health approach that emphasizes proactive, preventative, community-based interventions that target social determinants of health (e.g., poor social connectedness, financial concerns, relationship distress), thereby reducing risk of suicide [9, 24, 73]. Similarly, the Commander John Scott Hannon Act promises $174 million to efforts supporting community-based interventions to mitigate suicide risk among Veterans, particularly those who are not participating in VA care [74].

To aid in addressing the suicide risk and broader psychosocial needs of transitioning Servicemembers and Veterans (TSMVs), the Veteran Sponsorship Initiative (VSI) provides support to TSMVs throughout the transition process through connection to a trained, community-based peer sponsor. Consistent with recent efforts to maximize utility of public-private partnerships, the VSI is driven by operational partnerships between leaders of the VA, US Department of Defense (DoD), US Department of Labor, national nonprofit organizations, and community-based organizations. The current project represents the implementation of VSI at the state level.

### The Veteran Sponsorship Initiative as a Suicide Prevention Initiative

In recent years, theorists have used the Three-Step Theory of suicide (3ST; [36]) to understand TSMV suicide (e.g., [60]). Briefly, 3ST theorizes that (a) suicidal ideation results from a combination of pain and hopelessness, (b) connectedness is a key protective factor against increasing suicidal ideation severity, and (c) progression from suicidal ideation to attempt is moderated by the individual’s capability to end one’s life.

Consistent with the first tenant of 3ST, TSMVs often face significant psychological pain associated with reintegration into civilian life, such as that stemming from difficulties in education/employment, housing, food insecurity, health, relationship challenges, and legal involvement [11, 44, 55, 80]. Civilian employment, for example, is often cited as a significant reintegration concern; 43% of service members are not prepared to enter the civilian workforce as they exit the military [68]. Correspondingly, TSMVs often struggle to translate their military skillsets into a civilian setting, and many change jobs multiple times within the first few years of transition [41].

**Fig. 2:**
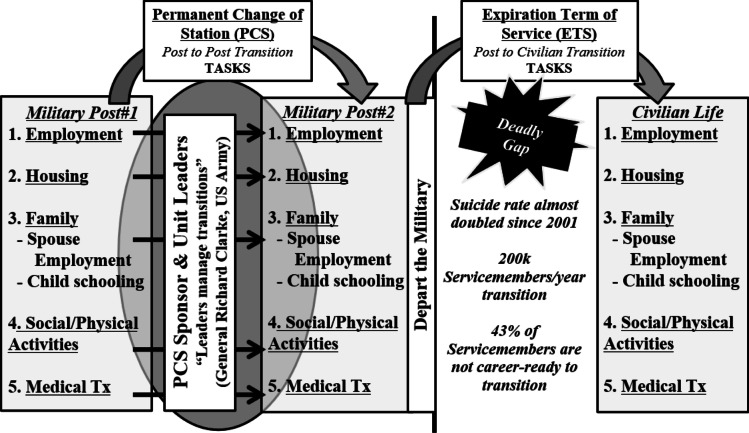
Transition Tasks and the Deadly Gap

Consistent with the second tenant of 3ST, many TSMVs also struggle to maintain connectedness—in relationships, values, purpose, and identity—throughout their transition [60]. While on active duty, the military promotes these forms of connectedness through specially selected and trained leaders (e.g., recruiters, drill sergeants, unit leaders). Each provides individualized support as servicemembers train with their units and deploy to combat. Indeed, research supports a positive association between leadership quality and mental health of combatants [7]. During times of transition, such as while conducting a permanent change of station (PCS) and moving from one military installation to another, the military also provides a PCS sponsor. For example, the U.S. Marine Corps has a thorough and required PCS sponsorship program that aims to reduce “stress and challenges associated with relocating” ([77], p. 2). PCS Sponsors are of equal rank and matched based on gender, marital status, and career field. They orient incoming servicemembers to their installation and help to accomplish transition tasks, such as establishing housing and community supports (see Fig. 2). Support received from such leaders is considered critical for servicemembers to accomplish their designated military missions [24]. By contrast, as TSMVs exit the military and reintegrate into civilian life, they rarely experience military support in their post-military destinations. Throughout this process, there are often fewer individuals, employers, family members, and colleagues to whom the TSMV can relate, and many report feeling closer to their military comrades than civilian counterparts after exiting the military [37].

Lastly, consistent with the final tenant of 3ST, military training and service increases capability for suicide through various routes. For example, military training, combat exposure, and increased risk of painful experiences associated with these experiences may increase acquired capability by desensitizing the TSMV to themes of death, injury, and pain [60]. Consistent with this, suicide rates are typically higher among Army and Marine Veterans, who have more explicit and provocative combat training [56]. Similarly, due to military culture, TSMVs are more likely than non-Veterans to own and have access to potentially lethal means, particularly firearms [12].

### The VSI structure

Informed by applications of 3ST to TSMVs, VSI aims to decrease TSMV suicide risk by decreasing psychological pain often associated with the military-to-civilian reintegration and increasing connectedness through pairing of TSMVs with trained, volunteer, community-based sponsors to support them throughout the transition process. Its structure and development were informed by recent legislation around public-private partnership approaches to suicide prevention; broader preventative, universal, and public health approaches to suicide prevention; the DoD’s PCS sponsorship program; and recommendations from the DoD’s *Best Practices Identified for Peer Support Programs* [67].

Briefly, the VSI consists of three core elements (see Fig. 1, Online Supplement):

- *Engage and enroll TSMVs on military installations*: TSMVs enroll in the VSI on their military installation approximately 6 months prior to discharge from active-duty military service. TSMVs are informed of the VSI during mandatory Transition Assistance Program (TAP) classes [69] and encouraged to enroll by their chain of command. Within seven days of VSI enrollment, TSMVs participate in an intake assessment with a VSI transition coordinator to identify reintegration needs and confirm their planned post-military destination.
- *Connect TSMVs with trained, certified sponsors*: Within 14 days of completing the intake assessment, TSMVs are matched with a sponsor and connected to a community integration coordinator (CIC) located in their planned post-military destination. After matching, TSMVs have regular contact with their sponsor through social media, email, and monthly video or in-person meetings. Contact continues until TSMVs are at least 6 months post-discharge and successfully reintegrated into their post-military destination.
- *Identify TSMV goals and needs and link with education/employment, healthcare, and other services*: During monthly sessions, sponsors help TSMVs to identify Specific-Measurable-Achievable-Relevant-Time Bound (SMART) goals related to domains of reintegration and social determinants of health (see Fig. 2; [25]). Within 30 days of matching, sponsors also assist in translating these goals into an individualized reintegration plan (i.e., “My Action Plan”). Sponsors and TSMVs then update the My Action Plan periodically through follow-up sessions based on the TSMV’s progress.

CICs are trained, local organizations (e.g., nonprofits, private hospitals, or county veteran service offices) that enter into the VSI public-private partnership. Primary responsibilities of the CIC include (a) recruiting volunteer sponsors and ensuring they attend VA certification training, (b) matching TSMVs with sponsors, (c) managing sponsors’ relationships with TSMVs in their local region, (d) submitting referrals to other agencies to assist TSMVs in meeting their goals, and (e) accessing and monitoring TSMV progress in the initiative using a common digital dashboard [75].

Because VA healthcare connection may be protective against Veteran suicide [74], CICs also ensure all eligible TSMVs are enrolled within VA healthcare and scheduled to attend a VA primary care appointment within 3 months post-discharge. Such an approach is consistent with recent research suggesting a key predictor of VA enrollment and utilization is VA engagement early in the military discharge process [78].

### Research supporting peer mentorship programs for TSMVs

An abundance of research suggests mentorship and sponsorship interventions may be beneficial to TSMVs. In a meta-analysis of 112 studies across a variety of samples, mentoring showed favorable associations with relational, career, and mental health outcomes [19]. Similarly, Veteran-based research attests to the value of mentoring-based interventions in educational, health care, and criminal justice contexts [2, 31, 82]. In an early pilot of VSI, Geraci and colleagues (Supporting transitioning service members and veterans using certified sponsors: A 3-arm randomized controlled trial, submitted) completed a randomized controlled trial in which 203 post-9/11 Veterans in the greater New York City area were randomly assigned to participate in either (a) a community-based Veteran social organization, (b) both a community-based Veteran social organization and VSI, or (c) a waitlist control condition. Results suggested Veterans participating in both a community-based Veterans social organization and VSI reported notably fewer reintegration difficulties and greater social connectedness (aligning to the first and second tenants of 3ST; [60]) across the course of their participation than Veterans in other conditions.

### Potential implications of the VSI

The current evaluation represents the first large-scale implementation of an evidence-based practice for TSMVs during the “deadly gap” of transition from military service to civilian life. Since establishment of the Foundations for Evidence-Based Policymaking Act (US PL 115-435) of 2018, VA must use evidence and evaluation to inform policies and budget allocations [29]. Therefore, the current evaluation has important implications for informing national implementation of community-based interventions to address the epidemic of TSMV suicide.

This evaluation is funded through a peer-reviewed Partnered Evaluation Initiative (PEI) grant provided by the VA Quality Enhancement Research Initiative (QUERI). PEI grants are a principal method of the VA to meet requirements of the Evidence-Based Policymaking Act. They focus on improving Veteran health by rapidly implementing evidence-based practices and planning for national scale-up and spread. Accordingly, this evaluation serves to implement the VSI throughout the state of Texas through a coordinated partnership between the VA QUERI, VA leadership within Texas (Veterans Integrated Services Network 17), and community partners. Texas has the second-largest population of Veterans aged 18 to 34 years old [76], which makes it an ideal setting for initial rollout of the VSI.

### Study aims

This evaluation is a hybrid type 2 effectiveness-implementation evaluation. The first aim of this evaluation is to assess the effectiveness of the VSI in improving proximal (reintegration difficulties, health/psychological distress, VA healthcare utilization, and connectedness) and distal (suicidal ideation and behaviors) factors associated with risk during the military-to-civilian transition. The second aim is to determine feasibility and utility of a bundled implementation strategy to expand the VSI to six cities in Texas (Austin, San Antonio, Houston, Dallas/Fort Worth, El Paso, and Corpus Christi; selected due to their proportionally large Veteran populations). Third, we intend to develop procedures to facilitate future expansion in other states. Design, implementation, and evaluation of the VSI is informed by the Reach, Effectiveness, Adoption, Implementation, and Maintenance framework (RE-AIM; [22]).

## Methods

### Evaluation framework and study design

This evaluation will use a stepped wedge design, which relies on sequential roll-out to participating cities over time, while using other cities as controls until they begin implementation. This design allows for both within-site and between-site comparisons of data. For within-site comparisons, each city will act as their own control by comparing data pre- versus post-implementation and throughout the transition from control (transition as usual; TAU) to intervention status. Between-site comparisons will compare TAU versus intervention cities. This combination of analyses will improve validity of the evaluation by minimizing statistical effects of historical trends occurring outside of the intervention and/or contextual characteristics that may impact an individual city’s performance.

**Fig. 3:**
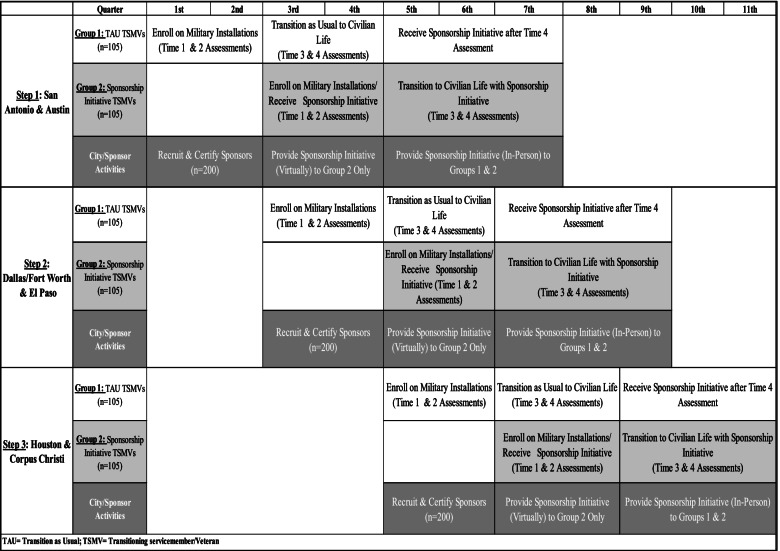
Stepped wedge design

The assignment of intervention status was randomized at the city level, and data from TSMVs will inform primary quantitative outcome analyses. Of the six cities participating in this evaluation, two cities were allocated to each of the three start dates (see Fig. 3). Because cities differ in local community and organization characteristics, we used the restricted selection method of randomization to balance cities across the three implementation steps over time [3] according to the number of projected TSMVs moving to each target location. Stratified randomization using methods described by Suresh [64] and computer programming were used for randomization procedures.

### TSMV participants and recruitment

To participate in VSI, TSMVs must be at least 18 years old, approximately 6 months from military discharge, and planning to transition to one of the evaluation’s target cities during an active enrollment window (see Fig. 3). TSMVs will be recruited from military installations across the USA. The first 105 TSMVs enrolled per step will be assigned to the TAU condition, and the next 105 TSMVs per step will be assigned to the VSI.

The sample size was determined by completing a statistical power analysis using the powerlmm package [40] for R [52]. To test the primary hypothesis—that VSI reduces reintegration difficulties—using multilevel modeling with power of 80%, alpha of .05, and a small effect size (Cohen’s *d*=0.2). Total enrolled TSMVs needed across the six cities is 630. This sample size allows for adjustments required under randomization, design effects, and a dropout rate of 20% per follow-up.

Potential sponsors will be recruited in target cities using social media outreach with the local VA, Veteran service organizations, and college and university alumni networks. In accordance with previous research, we anticipate a sponsor attrition rate of approximately 20%. To mitigate the risk of a shortage of sponsors, the VSI aims to certify at least 600 sponsors across the evaluation.

### Implementation strategies

**Fig. 4:**
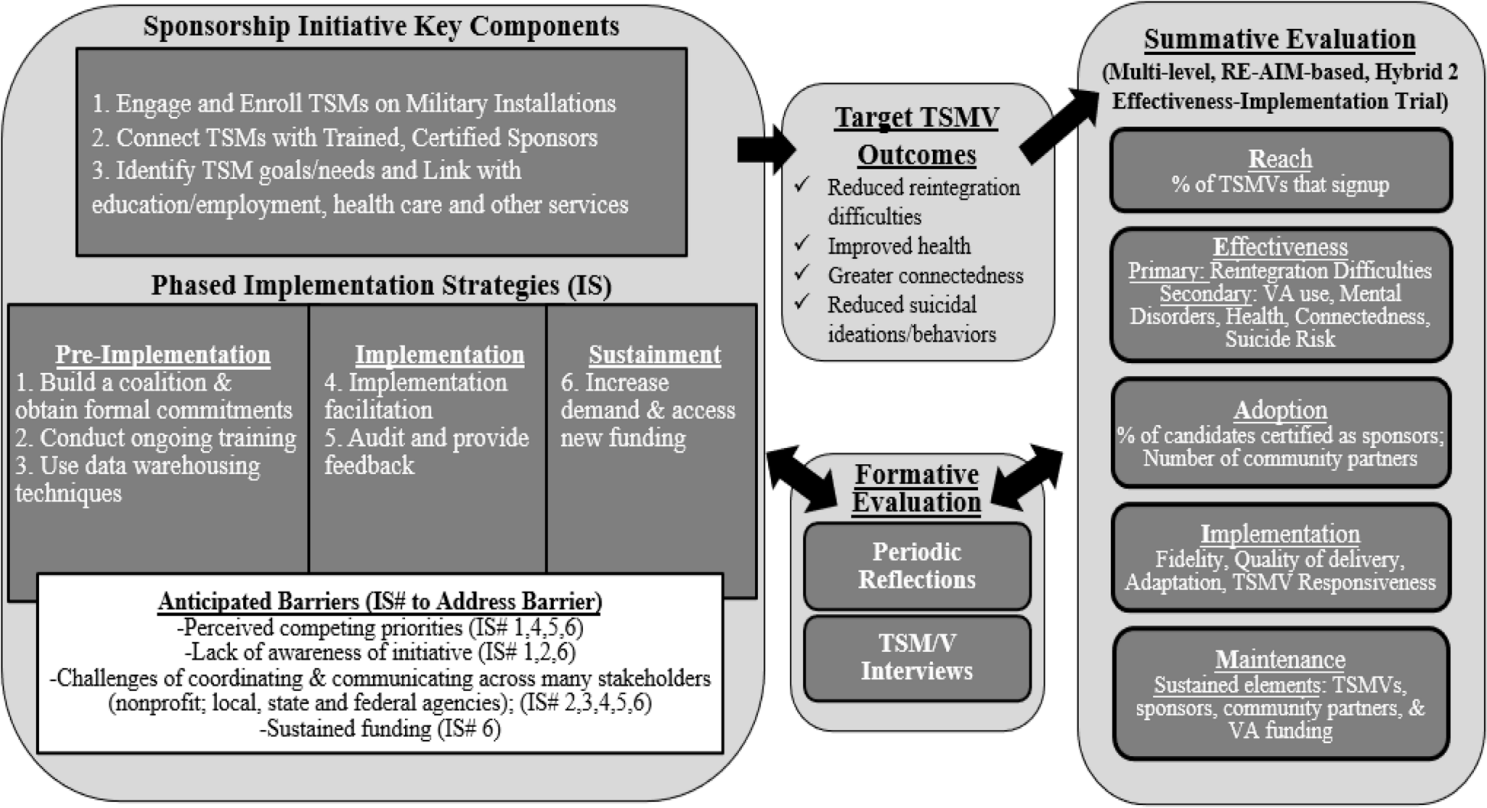
Overview of Veteran Sponsorship Initiative, Implementation Strategies and Evaluation Measures

A phased, bundled implementation strategy was developed according to the QUERI Implementation Roadmap [27, 34] and the Expert Recommendations for Implementing Change taxonomy [49] based on needs and barriers identified in an earlier pilot of the VSI. Strategies have been selected as appropriate to the unique challenges of the implementation phase—pre-implementation, implementation, and sustainment (see Fig. 4). As a pre-requisite to studying the implementation strategies empirically, the evaluators followed the reporting specifications provided by Proctor et al. [51] specific to naming, clearly describing, and operationalizing the respective implementation strategies (see Table 1, Online Supplement) with highlights provided below. We anticipate that the implementation strategies will assist in addressing the following barriers: (a) perceived competing priorities by local VA and community partners, (b) lack of awareness of the initiative, (c) challenges of coordinating and communicating across many stakeholders (nonprofit; local, state and federal agencies), and (d) need for sustained funding.

#### Pre-implementation

1. Build a coalition [49] and obtain formal commitments [49]. The VSI will identify and select CICs within each city based on predetermined criteria (see Table 1, Online Supplement). After selection, the VA will assist in the drafting and signing of a formal memorandum of agreement (MOA) between (1) selected CICs and (2) VA leadership within Texas (Veterans Integrated Services Network 17). MOAs will identify key responsibilities of each partner.
2. Conduct ongoing training [49]. The VA will train CICs, sponsors, VSI transition coordinators, and VA Regional Community Coordinators (VA RCCs) in accordance with the sponsor certification manual (see [25]) and the implementation toolkit. To support rapid expansion of the VSI and address challenges related to the COVID-19 pandemic, trainings will be offered in-person and virtually in each city.
3. Use data warehousing techniques [49]. The VSI will work collaboratively with community partners to develop referral workflows and plans for data warehousing. Appropriate authorizations will be secured and necessary procedures established to allow collected data to be utilized for operational and evaluation purposes. The VSI has worked with partners to ensure there is a  virtual dashboard to organize data and efforts by the CICs throughout this evaluation.

#### Implementation

4. Implementation facilitation [46]. The VA will hire and train VA RCCs to serve as external facilitators in Texas. These coordinators will be subject matter experts in assisting TSMVs throughout the military-to-civilian transition and will assist in implementation by developing an implementation toolkit and providing active support for the change process, including through audit and feedback activities with CICs. This toolkit will include guidebooks for (1) VA RCCs, (2) VSI Transition Coordinators, and (3) CICs. VA RCCs will also support change efforts, including identifying compatible workflows and create actionable plans to put best practices in place [46].
5. Audit and provide feedback [49]. Working with the evaluation team, the VA RCC will conduct assessments of performance of CICs and provide feedback from the assessments to CIC comparing their current status to prior time periods and a priori standards as benchmarks.

#### Sustainment

6. Increase demand [49] and Access new funding [49]. The evaluation team will develop quarterly reports and share them with VISN leadership in Texas, VA national leadership, local and state leadership, CICs in Texas, and potential CICs in other states. Within the reports, the team will highlight the status regarding VSI implementation and effectiveness variables. Following implementation, the evaluators will report on the overall budget impact analysis to support sustainment at the city level, as well as identify necessary resources to support initiative spread to other states. As part of the budget impact analysis, the evaluators will collect implementation and intervention cost data, including VA and non-VA materials, training costs, dashboard development/technology costs, CIC and transition coordinator personnel time and costs, and travel expenses. VHA healthcare utilization costs will be determined from accessing VHA administrative data on VHA cost and utilization to estimate the budget impact of implementing the VSI program using recommended approaches for budget impact analysis [63]. These health care costs will include VHA inpatient, outpatient, pharmacy, and VHA-paid community care costs with total costs estimated per TSMV per year and adjusted for inflation [17]. This will help to ensure sustainment in Texas and justify further allocation of funding for expansion into other states.

### RE-AIM

The RE-AIM framework (see Table 2, Online supplement) will allow the VSI to plan, assess, and adapt to the complexities resulting from a multi-organizational, collaborative endeavor across numerous locations. City-level analyses will examine RE-AIM measures before, during, and after implementation and facilitate formative and summative evaluation. *Reach* will be assessed by calculating the percent of eligible TSMVs who sign up for the VSI on respective military installations. *Effectiveness* will be assessed by TSMV-level data for proximal and distal variables (See Measures for more information). *Adoption* will be assessed by calculating the percent of sponsor candidates who complete certification and by counting the number of CICs who sign a VA Memorandum of Agreement.

*Implementation* will be assessed by four dimensions [5]: (a) *Fidelity* refers to adherence or program integrity and is the extent to which specified program components are delivered as prescribed [18]. To assess fidelity, we developed fidelity trackers that will assess to what extent core elements of the intervention are delivered as intended (see Table 2, Online Supplement); (b) *Quality of delivery* addresses the skill and behaviors with which sponsors deliver the VSI during their interactions with TSMVs. As part of the evaluation, TSMVs will complete an evaluation of their sponsor through the Leader-Behavior-Description-Questionnaire--Form XII [62] to assess the degree to which they perceive sponsors as engaging in relational-oriented and task-oriented leadership behaviors; (c) *Adaptation* concerns changes made to a program during implementation. Research attests to the value of allowing local communities to adapt programs according to the needs of a local population [47]. Adaptations will be documented and tracked using qualitative data from periodic reflections (see below). For this evaluation, we will (1) determine if observed changes stem from lack of fidelity or adaptation, and (2) categorize adaptations as either additions (i.e., activities or materials not part of the original initiative) or modification (i.e., activities or materials that were part of the original initiative but implemented in a way beyond prescribed variations). Evaluators will recommend sustainment and expansion of adaptations perceived as being effective and beneficial to TSMVs; (d) *Participant responsiveness* is identified as “levels of participation and enthusiasm” ([16], p. 45). We will measure participant responsiveness by calculating the number of sessions TSMV-sponsor pairs attend, percent of initial “My Action Plans” completed by TSMV-sponsor pairs within 30 days of matching, number of monthly updates made to action plans, and level of TSMV satisfaction with the VSI.

*Maintenance* will be assessed after the implementation phase and operationalized as the number of sponsors and CICs that remain engaged with the VSI, number of new sponsors registered, number of newly enrolled TSMVs, and continued VA funding dedicated to sustainment and expansion of the VSI.

### Measures

To facilitate evaluation of the VSI effectiveness, TSMVs will complete a range of online self-report measures and clinical interviews (Table 1; Fig. 2, Online Supplement). TSMVs will provide data at Time 1 (Baseline: 6 months prior to military discharge), Time 2 (2 months prior to military discharge), Time 3 (2 months post military discharge), and Time 4 (6 months post military discharge). To maximize clinical utility of data, many included measures are routinely administered at VA medical centers.

**Table 1:** Effectiveness variables

	Time 1 (6 months prior to military discharge)	Time 2 (2 months prior to military discharge)	Time 3 (2 months post military discharge)	Time 4 (6 months post military discharge)
**Step 1: Reduce psychological pain (proximal)**				
**A. Reintegration difficulties & SDOH**				
Military to Civilian Questionnaire (M2C-Q; [54])	X	X	X	X
Employment and Education Status	X	X	X	X
Income and Savings	X	X	X	X
Brief Resilience Scale (BRS; [59]).	X			X
VA Homelessness Screening Clinical Reminder:	X			X
US DA Food Security	X			X
Criminal Behaviors	X			X
Well-Being Items^a^	X			X
Qualitative Questions^a^ (What motivated you to sign up for the sponsorship initiative?; What do/did you hope will come out of being involved with the sponsorship initiative? Thinking about your transition out of military service, what are/have been your top three concerns?)	X			X
**B. Health (proximal)**				
Depression; Patient Health Questionnaire-9 [38]	X	X	X	X
Generalize Anxiety Disorder (GAD-7 [61];.	X	X	X	X
PC-PTSD-5 [50]	X	X	X	X
The Alcohol Use Disorders Identification Test–Consumption (AUDIT-C) [8]	X	X	X	X
Level of Personality Functioning - Brief Form 2.0 [81]	X	X	X	X
Somatic Symptom Scale 8 (SSS-8)	X	X	X	X
VA Healthcare Enrollment/Utilization^b^	X	X	X	X
**Step 2: Promote connectedness (proximal)**				
The Social Support Survey (MOS SSS) [58]	X	X	X	X
Qualitative Questions^a^. Thinking about your experience with the sponsorship initiative, what about the program has been most beneficial for you? What things about the sponsorship program have you found least beneficial or could be improved? Would you recommend this program to another Servicemember or Veteran? Why or why not?				X
**Suicidal ideation and behaviors (distal)**				
Columbia-Suicide Severity Rating Scale [33]^a^	X			X

^a^Interview

^b^Assessed through VHA medical records

### Reintegration difficulties and social determinants of health

*Reintegration difficulties* will be broadly assessed using the Military to Civilian Questionnaire (M2C-Q; [54]), a 16-item measure that assesses difficulties in (a) interpersonal relationships with family, friends, and peers; (b) productivity at work, school, or home; (c) community participation; (d) self-care; (e) leisure; and (f) perceived meaning in life. The M2C-Q was initially validated in a sample of Iraq and Afghanistan Veterans seeking VA healthcare services [54] and has since shown strong construct validity and internal reliability in numerous Veteran samples [10, 53].

*Employment/education and income/savings status* will be assessed by asking TSMVs to self-report the income of themselves and (if applicable) their spouse/domestic partner and other immediate family members; current educational enrollment status; current work status; job satisfaction; hours worked/salary over the last week for themselves and (if applicable) their spouse/domestic partner; and money set aside for financial emergency. TSMVs who are not currently employed or enrolled in school will be asked to report whether they are actively looking for paid employment and strategies used to find paid employment. Those not actively looking for paid employment will be asked to provide reasons for not looking for paid employment.

We will also analyze broader societal impacts of TSMV employment and education using common job training benefit methodologies (e.g., [4, 42]). For example, we will calculate aggregate increases in productivity attributable to TSMV employment stemming from VSI participation, long-term growth in productivity associated with TSMV higher education pursuit, potential increases in tax revenue for municipalities, and reductions in social welfare benefit payments. To project these aggregate income, tax revenue, and social welfare benefit changes, we will use point estimates from the evaluation coupled with estimates on typical variation in productivity and social welfare benefits from labor market entry.

*Resilience* will be assessed using the Brief Resilience Scale (BRS; [59]), a six-item (e.g., “I tend to bounce back quickly after hard times.”) scale with strong evidence of validity, test-retest reliability, and internal consistency across samples [84].

*Homelessness* will be assessed using the VA Homelessness Screening Clinical Reminder (HSCR; [43]), a four-item (e.g., “In the past two months, have you been living in stable housing that you own, rent, or stay in as part of a household?”) measure that assesses current homelessness or imminent risk of homelessness among Veterans. The HSCR is considered standard care within VA settings to identify and respond to instances of Veteran homelessness.

*Food insecurity* will be assessed using the U.S. Adult Food Security Survey Module [70], a three-stage screening measure for assessing food insecurity. An example question is: “Within the past 12 months, the food that I bought just didn’t last, and I didn’t have money to get more.” A response of “often” or “sometimes” indicates food insecurity.

*Criminal behaviors* will be assessed using a series of self-report items, including “Within the last 12 months have you been issued a ticket of any kind for a traffic violation (e.g., speeding, failure to signal)?”; “Within the last 12 months have you been arrested or charged for any type of criminal offense (e.g., DUI, disorderly conduct, drug offense, domestic violence, assault, robbery)?”; and “Within the last 12 months, has a restraining order, no contact agreement, or order of protection been initiated or taken against you?” Many studies have suggested self-report methods show good agreement with official records and are a generally valid means of assessing criminal behavior (e.g., [45]).

*Well-Being Signs* is a three-item screening measure, based on the work of Vogt et al. [79] used to assess how TSMVs are doing in their daily lives. It asks TSMVs to rate what percentage of time (from 0 to 100%) that they have been “Fully satisfied with how things are going in these aspects of life?”, “Regularly involved in all aspects of life that are important to you?”, and “Functioning your best in aspects of life that you do participate in?”.

### Health and healthcare utilization

*Mental health difficulties* will be assessed using the Patient Health Quesitonnaire-9 [38], Generalized Anxiety Disorder Questionnaire [61], Primary Care PTSD 5 [50], Alcohol Use Disorders Identification Test–Consumption [8], and Level of Personality Functioning Scale-Brief Form 2.0 [81]. These brief measures have demonstrated strong validity and reliability across samples [1, 13, 15, 32, 38, 50].

*Somatic symptom* burden related to stomach problems, back pain, headaches, chest pain, dizziness, energy, and sleep will be assessed by the Somatic Symptom Scale 8 [26]. Previous studies with Veterans demonstrated good item characteristics and excellent reliability, a sound factor structure and significant associations with related constructs like depression, anxiety, pain, quality of life, and impairment [65].

*VA healthcare enrollment and utilization* will be assessed through data in access to the VA Corporate Data Warehouse (CDW), which enables evaluators to determine rates of VHA enrollment and utilization. Utilization outcomes will include the number of VHA encounters per TSMVs per year for in-person and video telehealth primary care, mental health, specialty care, and emergency department visits, as well as in-patient and acute hospital stays (e.g., acute medical/surgical or psychiatric inpatient care; [28]). Utilization of VHA-sponsored care in the community will be obtained from community care data (Patient Integrity Tool data and Fee Basis data).

### Social connectedness

Connectedness will be assessed using the Medical Outcomes Study Social Support Survey [58], a 19-item self-report measure of perceived availability of social support or connectedness that has demonstrated strong psychometric properties in military [20], Veteran [14, 66], and civilian samples [58].

### Suicide ideation and behaviors

Suicidal ideation and behaviors will be assessed using questions from the Columbia Suicide Severity Rating Scale screener [48] supplemented by questions based on cues provided in the full screener about suicide attempts and injuries [33].

### Qualitative data collection

#### Semi-structured interviews

TSMVs will also participate in qualitative interviews at Time 1 and Time 4 to facilitate formative evaluation. At Time 1, TSMVs will provide information about their motivation and hopes for VSI enrollment and primary concerns prior to military discharge. At Time 4, they will provide information about their experience with the VSI, including strengths of VSI and areas for improvement.

#### Periodic reflections

To identify event, adaptations, and contextual factors impacting adoption, implementation, and maintenance, the evaluators will also integrate periodic reflections—an innovative, low-burden method for documenting implementation phenomena such as barriers, facilitators, and adaptations that occur during implementation [21]. These lightly structured and guided reflections will occur monthly with members of the evaluation team and quarterly with other key partners (e.g., sponsors, CICs, military installations). These reflections will increase qualitative data points throughout implementation, thereby increasing the likelihood of identifying and understanding sources of variation in implementation.

### Analyses

Multilevel models will be used to analyze quantitative data. TSMVs will be nested within each city when analyzing data collected during the specified timepoints. Multilevel random effects models examine within- and between-group change across time and by group (TAU vs. VSI). Multilevel modeling accounts for the underlying heterogeneity between and within participants (i.e., intercepts and slopes are allowed to vary across participants) and allows for identification of differences in rates of change (slopes) in the dependent variables between groups and controlling for confounding variables that may influence TSMV outcomes (e.g., gender). Multilevel models account for missing data by estimating the best fitting model from the data available for each participant [30]. Therefore, all data points for TSMVs who complete the pre-implementation assessment will be included in intent-to-treat (ITT) analyses.

During qualitative interviews, near-verbatim notes from TSMV interviews and periodic reflections with partners will be uploaded and maintained in Atlas.ti qualitative software, allowing for qualitative and mixed-method analyses. Qualitative thematic analysis will be used to identify the most frequently mentioned content areas. Independent coders will separate response text and conduct thematic analysis to identify topics occurring repeatedly (themes). Coders will independently review responses and hold meetings to finalize a list of key themes before systematically coding all responses.

## Discussion

As suicide rates for young and transitioning Veterans have increased, national efforts have emphasized preventative, universal, and public health approaches that embrace the value of public-private partnerships between government agencies and community organizations. As suggested by the 3ST, interventions that reduce pain associated with reintegration difficulties and improve connectedness may reduce TSMV risk for suicide [60]. By partnering with organizations already serving this high-risk subset of the Veteran population and incorporating sponsor-based programming, national suicide prevention efforts may be more effective.

This evaluation will have important implications for national implementation of community-based interventions to address the epidemic of TSMV suicide. Aligned with the Evidence-Based Policymaking Act, it is the first large-scale implementation of an evidence-based practice that supports TSMVs during the “deadly gap” of transition from military service to civilian life. The protocol outlined in this manuscript—a hybrid type 2 effectiveness-implementation evaluation—was developed in close partnership with relevant operations partners and will use state-of-the-art evaluation methods to answer key questions about how to best implement and sustain the VSI. A combination of quantitative and qualitative analyses will also allow for development of clear implementation and sustainment guidelines for operations partners.

## Supplementary Information

**Additional file 1: Figure 1.** Veteran Sponsorship Initiative: core elements, partnerships, and context. **Table 1.** Veteran Sponsorship Initiative Implementation Strategies (Per [51] Reporting Specifications). **Table 2.** RE-AIM summative evaluation. **Figure 2.** TSMV-level effectiveness variables.

## Data Availability

Not applicable, as this manuscript does not contain any data.

## Abbreviations

3ST: Three-Step Theory of Suicide
ETS: Expiration Term of Service
PEI: Partnered Evaluation Initiative Grant
QUERI: VA Quality Enhancement Research Initiative
TSMV: Transitioning Servicemember/Veteran
VA/VHA: US Department of Veterans Affairs/Veterans Health Administration
VSI: Veteran Sponsorship Initiative

## References

[1] Bach B, Simonsen S (2021). How does level of personality functioning inform clinical management and treatment? Implications for ICD-11 classification of personality disorder severity. Curr Opin Psychiatry.

[2] Barmak SA, Barmaksezian N, Der-Martirosian C (2021). Student veterans in higher education: the critical role of veterans resource centers. J Am Coll Heal.

[3] Bauer M, Miller C, Kim B, Lew R, et al. Partnering with health system operations leadership to develop a controlled implementation trial. Implement Sci. 2016. 10.1186/s13012-016-0385-7. 10.1186/s13012-016-0385-7 PMC4765154 26912342

[4] Belfield C, Nores M, Barnett S, Schweinhart L (2006). The high/scope perry preschool program: cost-benefit analysis using sata from the age-40 follow-up. J Hum Resour.

[5] Berkel C, Mauricio A, Schoenfelder E, Sandler I (2011). Putting the pieces together: an integrated model of program implementation. Prev Sci.

[6] Bernecker S, Zuromski K, Gutierrez P, Joiner T, King A, Liu H, et al. Predicting suicide attempts among soldiers who deny suicidal ideation in the army study to assess risk and resilience in servicemembers (Army STARRS). Behav Res Ther. 2019;120. 10.1016/j.brat.2018.11.018. 10.1016/j.brat.2018.11.018 PMC7594446 30598236

[7] Booth-Kewley S, Schmied EA, Highfill-McRoy RM, Larson GE, Garland CF, Ziajko LA (2013). Predictors of psychiatric disorders in combat Veterans. BMC Psychiatry.

[8] Bush K, Kivlahan DR, McDonell MB, Fihn SD, Bradley KA (1998). The AUDIT alcohol consumption questions (AUDIT-C): an effective brief screening test for problem drinking. Arch Intern Med.

[9] Carroll D, Kearney LK, Miller MA (2020). Addressing suicide in the Veteran population: engaging a public health approach. Front Psychiatry.

[10] Castillo EA, Mason J, D’Addario A, Chow GM, Tenenbaum G (2019). Resilience and Veteran community reintegration: an exploratory study. Mil Behav Health.

[11] Castro CA, Kintzle S, Hassan A. The state of the American veteran: The Los Angeles County Veterans study. University of Southern California; 2014. https://cir.usc.edu/wp-content/uploads/2013/10/USC010_CIRLAVetReport_FPpgs.pdf.

[12] Cleveland EC, Azrael D, Simonetti JA, Miller M (2017). Firearm ownership among American veterans: findings from the 2015 National Firearm Survey. Inj Epidemiol.

[13] Corson K, Gerrity MS, Dobscha SK (2004). Screening for depression and suicidality in a VA primary care setting: 2 items are better than 1 item. Am J Manag Care.

[14] Currier JM, Lisman R, Irene Harris J, Tait R, Erbes CR (2013). Cognitive processing of trauma and attitudes toward disclosure in the first six months after military deployment. J Clin Psychol.

[15] Currier JM, Holland JM, Drescher K, Foy D (2015). Initial psychometric evaluation of the Moral Injury Questionnaire—Military version. Clin Psychol Psychother.

[16] Dane AV, Schneider BH (1998). Program integrity in primary and early secondary prevention: are implementation effects out of control?. Clin Psychol Rev.

[17] Dunn A, Grosse SD, Zuvekas SH (2018). Adjusting health expenditures for inflation: a review of measures for health services research in the united states. Health Serv Res.

[18] Durlak J, DuPre E (2008). Implementation matters: a review of research on the influence of implementation on program outcomes and the factors affecting implementation. Am J Community Psychol.

[19] Eby L, Allen TD, Hoffman BJ (2013). An interdisciplinary meta-analysis of the potential antecedents, correlates, and consequences of protégé perceptions of mentoring. Psychol Bull.

[20] Erbes CR, Kramer M, Arbisi PA, DeGarmo D, Polusny MA (2017). Characterizing spouse/partner depression and alcohol problems over the course of military deployment. J Consult Clin Psychol.

[21] Finley EP, Huynh AK, Farmer MM, Bean-Mayberry B, Moin T, Oishi SM, Moreau JL, Dyer KE, Landam HJ, Leykum L, Hamilton AB (2018). Periodic reflections: a method of guided discussions for documenting implementation phenomena. BMC Med Res Methodol.

[22] Gaglio B, Shoup JA, Glasgow RE (2013). The RE-AIM framework: a systematic review of use over time. Am J Public Health.

[23] Geraci J, Murray C, Kapil-Pair KN, Herrera S, Sokol Y, Cary J, Landa Y, Goodman M (2020). The modern-day Odysseus: how mental health providers can better reintegrate modern warriors and mitigate suicide risk. J Clin Psychol.

[24] Geraci J, Mobbs M, Edwards E, Goodman M (2020). Expanded roles and recommendations for stakeholders to successfully reintegrate modern warriors and mitigate suicide risk. Front Psychol.

[25] Geraci J, Kilby D, Arenz J (2020). Trained, peer sponsorship/mentorship training manual. VISN 2 MIRECC.

[26] Gierk B, Kohlmann S, Kroenke K (2014). The somatic symptom scale–8 (SSS-8): a brief measure of somatic symptom burden. JAMA Intern Med.

[27] Goodrich DE, Miake-Lye I, Braganza MZ, Wawrin N, Kilbourne AM (2020). Quality enhancement research initiative roadmap for implementation and quality improvement.

[28] Gujral K, Scott J, Ambady L, Dismuke-Greer C, Jacobs J, Chow A, et al. A primary care telehealth pilot program to improve access: associations with patients’ health care utilization and costs. Telemed J E Health. 2021;28(5):643–53. Advanced online. 10.1089/tmj.2021.0284 34559017

[29] Hahn R (2019). Building upon foundations for evidence-based policy. Science.

[30] Hedeker D, Gibbons RD (2006). Longitudinal data analysis.

[31] Jalain CI, Grossi EL (2020). Take a load off fanny: peer mentors in Veterans Treatment Courts. Crim Justice Policy Rev.

[32] Jordan P, Shedden-Mora MC, Löwe B. Psychometric analysis of the Generalized Anxiety Disorder scale (GAD-7) in primary care using modern item response theory. PLoS One. 2017;12(8). 10.1371/journal.pone.0182162. 10.1371/journal.pone.0182162 PMC5542568 28771530

[33] Katz I, Barry CN, Cooper SA, Kasprow WJ, Hoff RA (2019). Use of the Columbia-Suicide Severity Rating Scale (C-SSRS) in a large sample of Veterans receiving mental health services in the Veteran Health Administration. Suicide Life Threat Behav.

[34] Kilbourne AM, Goodrich DE, Miake-Lye I, Braganza MZ, Bowersox NW (2019). Quality enhancement research initiative implementation roadmap: toward sustainability of evidence-based practices in a learning health system. Med Care.

[35] Kline A, Ciccone DS, Falca-Dodson M (2011). Suicidal ideation among National Guard troops deployed to Iraq. J Nerv Ment Dis.

[36] Klonsky ED, May AM (2015). The three-step theory (3ST): A new theory of suicide rooted in the “ideation-to-action” framework. Int J Cogn Ther.

[37] Koenig CJ, Maguen S, Monroy JD, Mayott L, Seal KH (2014). Facilitating culture-centered communication between health care providers and Veterans transitioning from military deployment to civilian life. Patient Educ Couns.

[38] Kroenke K, Spitzer RL, Williams JB, Löwe B (2010). The patient health questionnaire somatic, anxiety, and depressive symptom scales: a systematic review. Gen Hosp Psychiatry.

[39] Louzon S, Bossarte R, McCarthy J, Katz I (2016). Does Suicidal Ideation as Measured by the PHQ-9 Predict Suicide Among VA Patients?. Psychiatr Serv.

[40] Magnusson K (2018). powerlmm: power analysis for longitudinal multilevel models. (Version 0.4.0.) [Computer software].

[41] Maury R, Stone B, Roseman J. Veteran job retention survey. Institute for Veterans and Military Families and Vet Advisor. Syracuse; 2016.

[42] McConnell S, Glazerman S (2001). National jobs study: the benefits and costs of job corps.

[43] Montgomery AE. Using a universal screener to identify Veterans experiencing housing instability. VA National Center on Homelessness Among Veterans; 2014. Retrieved from https://www.va.gov/HOMELESS/nchav/resources/docs/prevention/Homeless-Screener/Using-a-Universal-Screener-to-Identify-Veterans-Experiencing-Housing-Instability-508.pdf.

[44] Morin R (2011). The difficult transition from military to civilian life.

[45] Morris NA, Slocum LA (2010). The validity of self-reported prevalence, frequency, and timing of arrest: An evaluation of data collected using a life event calendar. J Res Crime Delinq.

[46] Perry C, Damschroder L, Hemler J, Woodson T, Ono S, Cohen D. Specifying and comparing implementation strategies across seven large implementation interventions: a practical application of theory. Implement Sci. 2019;(14):32. 10.1186/s13012-019-0876-4. 10.1186/s13012-019-0876-4 PMC6429753 30898133

[47] Palinkas LA, Aarons GA, Chorpita BF, Hoagwood K, Landsverk J, Weisz JR (2009). Cultural exchange and the implementation of evidence-based practices: two case studies. Res Soc Work Pract.

[48] Posner K, Brown GK, Stanley B, Brent DA, Yershova KV, Oquendo MA, Currier GW, Melvin GA, Greenhill L, Shen S, Mann JJ (2011). The Columbia-Suicide Severity Rating Scale: initial validity and internal consistency findings from three multisite studies with adolescents and adults. Am J Psychiatry.

[49] Powell BJ, Waltz TJ, Chinman MJ, Damschroder LJ, Smith JL, Matthieu MM, Proctor EK, Krichner JE. A refined compilation of implementation strategies: results from the Expert Recommendations for Implementing Change (ERIC) project. Implementation Sci. 2015;10(21). 10.1186/s13012-015-0209-1. 10.1186/s13012-015-0209-1 PMC4328074 25889199

[50] Prins A, Bovin MJ, Smolenski DJ, Marx BP, Kimerling R, Jenkins-Guarnieri MA, Kaloupek DG, Schnurr PP, Kaiser AP, Leyva YE, Tiet QQ (2016). The Primary Care PTSD Screen for DSM-5 (PC-PTSD-5): development and evaluation within a Veteran primary care sample. J Gen Intern Med.

[51] Proctor E, Powell B, McMillen J (2013). Implementation strategies: recommendations for specifying and reporting. Implement Sci.

[52] R Core Team R (2020). A language and environment for statistical computing. (Version 4.0.3) [Computer software].

[53] Romaniuk M, Fisher G, Kidd C, Batterham PJ (2020). Assessing psychological adjustment and cultural reintegration after military service: development and psychometric evaluation of the post-separation military-Civilian Adjustment and Reintegration Measure (M-CARM). BMC Psychiatry.

[54] Sayer N, Frazier P, Orazem R (2011). Military to civilian questionnaire. J Trauma Stress.

[55] Sayer NA, Orazem RJ, Noorbaloochi S, Gravely A, Frazier P, Carlson KF, Schnurr PP, Oleson H (2014). Iraq and Afghanistan war Veterans with reintegration problems: differences by Veterans Affairs healthcare user status. Adm Policy Ment Health Ment Health Serv Res.

[56] Scoville SL, Gubata ME, Robert N, Potter RN, White MJ, Pearse LA. Deaths Attributed to Suicide among Enlisted U.S. Armed Forces Recruits, 1980–2004. Mil Med. 2007;172(10):1024–31. 10.7205/MILMED.172.10.1024. 10.7205/milmed.172.10.1024 17985760

[57] Shen YC, Cunha JM, Williams T (2016). Time-varying associations of suicide with deployments, mental health conditions, and stressful life events among current and former US military personnel: a retrospective multivariate analysis. Lancet Psychiatry.

[58] Sherbourne CD, Stewart AL (1991). The MOS social support survey. Soc Sci Med.

[59] Smith BW, Dalen J, Wiggins K, Tooley E, Christopher P, Bernard J (2008). The brief resilience scale: assessing the ability to bounce back. Int J Behav Med.

[60] Sokol Y, Gromatsky M, Edwards E, Greene A, Geraci J, Harris R, et al. The deadly gap: understanding suicide among Veterans transitioning out of the military. Psychiatry Res. 2021;300. 10.1016/j.psychres.2021.113875. 10.1016/j.psychres.2021.113875 33901974

[61] Spitzer RL, Kroenke K, Williams JBW, Lowe B (2006). A brief measure for assessing generalized anxiety disorder. Arch Intern Med.

[62] Stogdill RM (1963). Manual for the Leader Behaviour Description Questionnaire-Form XII: an experimental revision.

[63] Sullivan SD, Mauskopf JA, Augustovski F (2014). Principles of good practice for budget impact analysis II: report of the ISPOR Task Force on Good Research Practices – Budget Impact Analysis. Value Health.

[64] Suresh KP (2011). An overview of randomization techniques: an unbiased assessment. J Hum Reprod Sci.

[65] Toussaint A, Kroenke K, Baye F, Lourens S (2017). Comparing the patient health 1uestionnaire - 15 and the somatic symptom scale - 8 as measures of somatic symptom burden. J Psychosom Res.

[66] Tsai J, Snitkin M, Trevisan L, Kraus SW, Pietrzak RH (2019). Awareness of suicide prevention programs among US military Veterans. Adm Policy Ment Health Ment Health Serv Res.

[67] US Department of Defense. Best practices identified for peer support programs. Washington D.C.: Defense Centers of Excellence for Psychological Health & Traumatic Brain Injury; 2011.

[68] US Department of Defense (2021). Evaluation of the department of defense’s implementation of suicide prevention resources for transitioning uniformed service members.

[69] US Department of Defense (2022). DoD transition assistance program.

[70] US Department of Agriculture (2012). Adult food security survey module: three-stage design.

[71] US Department of Veterans Affairs. The military to civilian transition 2018. Washington, DC; 2018a. Retrieved from https://benefits.va.gov/TRANSITION/docs/mct-report-2018.pdf

[72] US Department of Veterans Affairs (2018). Profile of post-9/11 veterans: 2016.

[73] US Department of Veterans Affairs (2018). National strategy for preventing Veteran suicide, 2018–2028.

[74] US Department of Veterans Affairs (2021). Annual report- National Veteran suicide prevention.

[75] US Department of Veterans Affairs (2022). Veteran sponsor partnership network.

[76] US Department of Veterans Affairs (2022). Veteran population: the states.

[77] US Navy. U.S. Marine Corps Sponsorship Program. Washington D.C.: Department of Defense; 2012.

[78] Vanneman ME, Harris AHS, Chen C, Mohr BA, Adams RS, Williams TV, Larson MJ (2015). Army active-duty members’ linkage to Veterans Health Administration services after deployments to Iraq or Afghanistan and following separation. Mil Med.

[79] Vogt D, King MW, Borowski S, Finely EP, Perkins DF, Copeland LA. Identifying factors that contribute to military veterans’ post-military well-being. Appl Psychol Health Well-Being. 2021;13(2):341–56. https://doi-org.ezproxy.cul.columbia.edu/10.1111/aphw.12252. 10.1111/aphw.12252 33595207

[80] Vogt DS, Tyrell FA, Bramande EA, Nillni YI, Taverna EC, Finely EP, Perkins DF, Copeland LA (2020). U.S. military Veterans’ health and well-being in the first year after service. Am J Prev Med.

[81] Weekers LC, Hutsebaut J, Kamphuis JH (2019). The level of personality functioning scale-Frief form 2.0: update of a brief instrument for assessing level of personality functioning. Personal Ment Health.

[82] Weir B, Cunningham M, Abraham L, Allanson-Oddy C (2019). Military veteran engagement with mental health and well-being services: a qualitative study of the role of the peer support worker. J Ment Health.

[83] White House (2021). Reducing military and Veteran suicide: advancing a comprehensive, cross-sector, evidence-informed public health strategy.

[84] Windle G, Bennett KM, Noyes J (2011). A methodological review of resilience measurement scales. Health Qual Life Outcomes.

