# Supplementary material for: Partnered implementation of the veteran sponsorship initiative: protocol for a randomized hybrid type 2 effectiveness—implementation trial
Source: Implement Sci. 2022 Jul 8;17:43. doi: 10.1186/s13012-022-01212-9 (PMC9264302; doi:10.1186/s13012-022-01212-9)
Supplement: Supplementary file 1 — Additional file 1: Figure 1. Veteran Sponsorship Initiative: core elements, partnerships, and context. Table 1. Veteran Sponsorship Initiative Implementation Strategies (Per [51] Reporting Specifications). Table 2. RE-AIM summative evaluation. Figure 2. TSMV-level effectiveness variables. [file 13012_2022_1212_MOESM1_ESM.docx]

**Online Supplement**


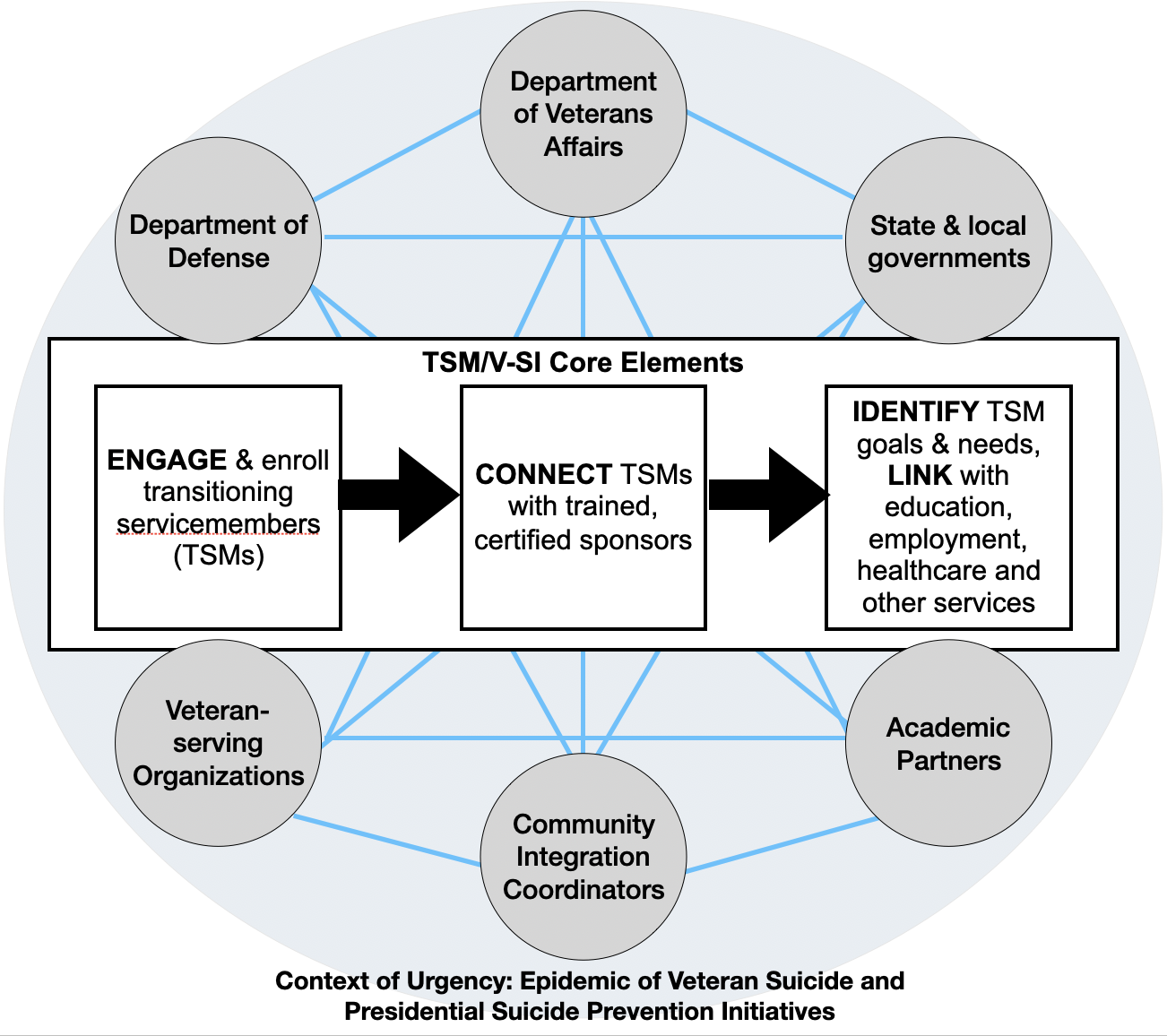


Figure 1. Veteran Sponsorship Initiative: Core Elements, Partnerships, and Context

**Table 1. Veteran Sponsorship Initiative Implementation Strategies (Per Proctor et al., 2013 Reporting Specifications)**

| **Reporting Specifications** | **Description** |
| --- | --- |
| **1. Name** | **1. Build a coalition (Powell et al., 2015) and obtain formal commitments (Powell et al., 2015)** |
| **2. Definition** | “Build a coalition. Recruit and cultivate relationships with partners in the implementation effort” (Powell et al., 2015, p. 8)  “Obtain formal commitments. Obtain written commitments from key partners that state what they will do to implement the innovation” (Powell et al., 2015, p. 9) |
| **3. Operationalize it** | |
| **Actor(s)** | The Expiration Term of Service Sponsorship Program (ETS-SP) and VA Healthcare Advancement and Partnerships (VA HAP) |
| **Action(s)** | Community integration coordinators (CICs) are trained, local organizations (e.g., nonprofits, private hospitals, or county veteran service offices) that enter into the Veteran Sponsorship Initiative public-private partnership. Primary responsibilities of the CIC include: (a) recruiting volunteer sponsors and ensuring they attend VA certification training; (b) matching transitioning Servicemembers/Veterans (TSMVs) with sponsors; (c) managing sponsors’ relationships with TSMVs in their local region (d) submitting referrals to other agencies to assist TSMVs in meeting their goals and (e) accessing and monitoring TSMV progress in the initiative using a common digital dashboard. ETS-SP will identify and select CICs within each city based on the following criteria as described in CIC applications: A. Overview (30%; organization philosophy, principal activities, city/state grant experience, services provided to TSMVs and experience managing a volunteer and mentor/sponsor population), B. Proposed Service Delivery (40%; staffing plan and external partners), and C. Funding Mechanisms (30%; plan and sustainability to fund actions; capabilities to capture costs). After selection, the VHA HAP will assist in the drafting and signing of a formal memorandum of agreement (MOA) between: 1. ETS Sponsorship, 2. The selected CIC, and 3. VA leadership within Texas (Veterans Integrated Services Network 17). MOAs will identify key responsibilities of each partner. |
| **Target(s) of the action** | CICs in respective cities |
| **Temporality** | MOAs will be created in the pre-implementation phase for each respective city |
| **Dose** | MOAs will be created with each CIC per city during the pre-implementation phase. Additional CICs and MOAs will be developed, as necessary, throughout the implementation process per city. |
| **Implementation outcome(s) affected** | Adoption: Number of CICs who become actively involved and sign a VA Memorandum of Agreement |
| **Justification** | Building a coalition and obtaining formal commitments helps to clarify roles, responsibilities, detail tangible and non-tangible benefits and ensure that key roles are supported by the partners (QUERI, 2020). |

| **Reporting Specifications** | **Description** |
| --- | --- |
| **1. Name** | **2. Conduct ongoing training (Powell et al., 2015)** |
| **2. Definition** | “Plan for and conduct training in the clinical innovation in an ongoing way for all individuals involved with implementation and users of the clinical innovation (e.g., clinicians, implementation staff, practice facilitators)” (Perry et al., 2019, p. 6) |
| **3. Operationalize it** | |
| **Actor(s)** | VA Transitioning Servicemember/Veteran And Suicide Prevention Center (VA TASC) |
| **Action(s)** | The VA TASC will train CICs, sponsors, VSI transition coordinators and VA Regional Community Coordinators (VA RCCs) in accordance with the sponsor certification manual (see Geraci et al., under review and Geraci et al., 2020c) and the implementation toolkit. To support rapid expansion of the VSI and address challenges related to the COVID-19 pandemic, trainings will be offered in-person and virtually in each city. |
| **Target(s) of the action** | CICs, sponsors, VSI transition coordinators and VA RCCs |
| **Temporality** | Training will occur in the pre-implementation phase for each respective city |
| **Dose** | Trainings range from 3 hours for CIC training to 7.5 hours for sponsorship training. |
| **Implementation outcome(s) affected** | Adoption: percent of sponsor candidates who sign up for VSI and then become certified  Fidelity: extent to which the core elements of the intervention are delivered as intended (Core elements: 1. Engage and Enroll TSMVs on Military Installations; 2. Connect TSMVs with Trained, Certified Sponsors; 3. Identify TSMV goals/needs and Link with education/employment, healthcare and other services)  Quality of delivery: TSMV responses on the Leader Behaviour Description Questionnaire-Form XII (Stogdill, 1963) |
| **Justification** | This is the first time that many of the CICs, sponsors, VSI transition coordinators and VA RCCs have conducted their respective responsibilities, as part of the VSI. Ensuring that all entities receive standardized and high-quality training with help improve the implementation outcomes of adoption, fidelity and quality of delivery. |

| **Reporting Specifications** | **Description** |
| --- | --- |
| **1. Name** | **3. Use data warehousing techniques (Powell et al., 2015)** |
| **2. Definition** | “Integrate clinical records across facilities and organizations to facilitate  implementation across systems” (Powell, 2015, p. 10). |
| **3. Operationalize it** | |
| **Actor(s)** | ETS-SP, VA TASC and VA Office of Information & Technology (VA OI&T) |
| **Action(s)** | The VSI will work collaboratively with community partners to develop referral workflows and plans for data warehousing. Appropriate authorizations will be secured and necessary procedures established to allow collected data to be utilized for operational and evaluation purposes. ETS-SP has developed a virtual dashboard to organize data and efforts by the CICs throughout this evaluation. |
| **Target(s) of the action** | Sponsors and CICs |
| **Temporality** | Weekly meetings will occur with ETS-SP, VA TASC, and VA OI&T to coordinate data warehousing efforts throughout the pre-implementation and implementation phases |
| **Dose** | Weekly meetings will last for 1 hour |
| **Implementation outcome(s) affected** | Fidelity: extent to which the core elements of the intervention are delivered as intended (1. Engage and Enroll TSMVs on Military Installations; 2. Connect TSMVs with Trained, Certified Sponsors;  3. Identify TSMV goals/needs and Link with education/employment, healthcare and other services) |
| **Justification** | A key feature of implementation is the linking of local data to data platforms outside the local setting, such as through a central data repository. Such warehousing will allow for improved communication and coordination across partners and agencies, allowing for timely and efficient sponsor/TSVM matching and service linkages as needed, offering benchmarking capability, and facilitating audit and feedback (QUERI, 2020; Perry et al., 2019). |

| **Reporting Specifications** | **Description** |
| --- | --- |
| **1. Name** | **4. Implementation Facilitation (Perry et al., 2019)** |
| **2. Definition** | “A multi-faceted interactive process of problem solving, enabling and supporting individuals, groups and organizations in their efforts to adopt and incorporate innovations into routine practices that occurs in a context of a recognized need for improvement and a supportive interpersonal relationship” (Perry et al., 2019, p. 5) |
| **3. Operationalize it** | |
| **Actor(s)** | VA Regional Community Coordinators (VA RCCs) |
| **Action(s)** | The VA will hire and train VA RCCs to serve as external facilitators in Texas. These coordinators will be subject matter experts in assisting TSMVs throughout the military-to-civilian transition and will develop and share key implementation materials and resources (e.g., guidebooks by role) and assisting in change process efforts (Perry et al., 2019). |
| **Target(s) of the action** | CICs and local VA medical center staff |
| **Temporality** | Monthly feedback meetings will occur with CICs in respective cities after that city enters the pre-implementation phase, as described in the stepped wedge design. The meetings will continue throughout the implementation phase. VA RCCs will also conduct in-person and virtual meetings with CICs and local VA medical staff at a frequency deemed most appropriate during the pre-implementation phase. |
| **Dose** | Monthly feedback meetings will be one hour in duration. The duration of additional in-person and virtual meetings between VA RCCs, CICs and local VA medical staff will be determined during the pre-implementation phase. They will occur during the pre-implementation and implementation phases. |
| **Implementation outcome(s) affected** | Fidelity: extent to which the core elements of the intervention are delivered as intended (1. Engage and Enroll TSMVs on Military Installations; 2. Connect TSMVs with Trained, Certified Sponsors;  3. Identify TSMV goals/needs and Link with education/employment, healthcare and other services) |
| **Justification** | Implementation facilitation has been used to support the implementation of interventions and to facilitate sites accomplishing their implementation goals (Perry et al., 2019). |

| **Reporting Specifications** | **Description** |
| --- | --- |
| **1. Name** | **5. Audit and provide feedback (Powell et al., 2015)** |
| **2. Definition** | “Develop summaries of clinical performance over a specific time period, often including a comparator, and give it to clinicians and/or administrators. Summary content (e.g., nature of the data,  choice of comparator) and their delivery (e.g., mode, format) are designed to modify specifically targeted behavior(s) or actions of individual practitioners, teams, or health care organizations” (Perry et al., 2019, p. 5). |
| **3. Operationalize it** | |
| **Actor(s)** | VA Regional Community Coordinators (VA RCCs) |
| **Action(s)** | Working with the evaluation team, the VA RCC will conduct assessments of performance of CICs and provide feedback from the assessments to CIC comparing their current status to prior time periods and a priori standards as benchmarks. |
| **Target(s) of the action** | Audit and provide feedback is intended to modify targeted behaviors of CICs |
| **Temporality** | Monthly feedback meetings will occur with CICs in respective cities after that city enters the pre-implementation phase, as described in the stepped wedge design. The meetings will continue throughout the implementation phase. |
| **Dose** | Monthly feedback meetings will be one hour in duration. |
| **Implementation outcome(s) affected** | Fidelity: extent to which the core elements of the intervention are delivered as intended (1. Engage and Enroll TSMVs on Military Installations; 2. Connect TSMVs with Trained, Certified Sponsors;  3. Identify TSMV goals/needs and Link with education/employment, healthcare and other services) |
| **Justification** | Audit and provide feedback is a widely used strategy as a component of multifaceted quality improvement interventions and can lead to important improvements in professional practice (Ivers, 2012). |

| **Reporting Specifications** | **Description** |
| --- | --- |
| **1. Name** | **6. Increase demand (Powell, 2015) and Access new funding (Powell, 2015).** |
| **2. Definition** | “Increase demand. Attempt to influence the market for the clinical innovation to increase competition intensity and to increase the maturity of the market for the clinical innovation” (Powell, 2015, p. 9).  “Access new funding. Access new or existing money to facilitate the implementation” (Powell, 2015, p. 8). |
| **3. Operationalize it** | |
| **Actor(s)** | **Evaluation team, VA HAP, ETS-SP and VA RCCs** |
| **Action(s)** | Quarterly reports: The evaluation team will develop quarterly reports and share them with VISN leadership in Texas, VA national leadership, local and state leadership, CICs in Texas and potential CICs in other states. Within the reports, the team will highlight the status regarding VSI implementation and effectiveness variables.  Conduct and disseminate budget impact analysis: The evaluators will also highlight the overall budget impact analysis to support sustainment at the city level, as well as identifying necessary resources to support initiative spread to other states. This will help to ensure sustainment in Texas and justify further allocation of funding for further expansion into other states. |
| **Target(s) of the action** | VISN leadership in Texas, VA national leadership, local and state leadership, CICs in Texas and potential CICs in other states |
| **Temporality** | Quarterly reports will initiate three months following implementation launch in each site; budget impact analysis will be conducted six months following implementation and be delivered to partners in late implementation to aid in sustainment planning |
| **Dose** | Once a quarter for quarterly reports; one-time for budget impact analysis |
| **Implementation outcome(s) affected** | Maintenance:  -Continued funding dedicated to VSI sustainment and expansion |
| **Justification** | In order for the VSI to continue within Texas beyond the implementation and for the VSI to expand to other states, it will be necessary to continue to increase demand for the VSI and to access new funding. |

**Table 2. RE-AIM Summative Evaluation.**

|  | **Description/ Definition** | **Information** |  |
| --- | --- | --- | --- |
| **Reach** | “How do I reach the targeted population with the intervention?” | - Percent of eligible TSMVs who sign up for the VSI compared to the total number of eligible TMSVs on respective military installations. |  |
| **Effectiveness** | “How do I know my intervention is effective for target population?” | Proximal Variables:  *Step 1: Reduce Psychological Pain*   1. Reintegration Difficulties & SDOH: Employment/education status, income/savings, resilience, crime, homelessness, food security 2. Health: VA enrollment and utilization, depression, anxiety, alcohol use, insomnia, pain.   *Step 2: Connectedness*  Distal Variables:  -Suicidal ideation and behaviors |  |
|  |  |  |  |
|  |  |  |  |
|  |  |  |  |
| **Adoption** | “How do I develop organizational support to deliver my intervention?” | - Percent of sponsor candidates who sign up for the VSI and then become certified. - Number of CICs who become actively involved and sign a VA Memorandum of Agreement– Qualitative data will describe barriers and facilitations to adoption |  |
|  |  |  |  |
|  |  |  |  |
|  |  |  |  |
| **Implementation** | “How do I ensure the intervention is delivered properly?” | **Fidelity:** extent to which the core elements of the intervention are delivered as intended  1. Engage and Enroll TSMVs on Military Installations  -Enroll TSMVs 6 months prior to their discharge from the military on their active-duty installation  -Transition Coordinators conduct intake within 7 days of enrollment  2. Connect TSMVs with Trained, Certified Sponsors  -CICs match TSMVs with sponsors within 14 days of intake  -Sponsors have regular contact with their TSMs via social media/email and conduct monthly video or in-person sessions for 12 months  3. Identify TSMV goals/needs and Link with education/employment, healthcare and other services  -TSMs create initial “My Action Plan” with sponsors within 30 days of matching  -TSMs update “My Action Plan” on a monthly basis  -CICs receive and process referrals for all TSMVs’ goals/needs within 7 days of identified on TSMVs’ “My Action Plans”  -Transition Coordinators enroll TSMVs in VHA (with priority code) within 30 days post-discharge  -CICs ensure TSMVs attend a VHA primary care appointment within 3 months post-discharge, if eligible  -CICs, Transition Coordinators and sponsors refer all TSMVs who screen + for acute risk to care within 24 hours  **Qualitative data from periodic reflections will inform understandings of fidelity to core program elements and factors impacting fidelity and adaptation  **Quality of delivery:** TSMVs will complete an evaluation of their sponsor through the Leader-Behavior-Description-Questionnaire-LBDQ (Halpin, 1957), that assesses the frequency with which TSMVs perceive their sponsors engage in two types of leadership behaviors: relational-oriented (“is friendly and approachable”) and task-oriented (“emphasizes the meeting of deadlines”). Items are rated on a 5-point Likert scale [1 (never) to 5 (always)]  **Adaptation**:  -We will first distinguish if an observed change is indicative of a lack of fidelity or an adaptation.  -Adaptations will be categorized as either an addition (e.g., activity or material that was not part of the original program) or a modification (e.g., activities that were part of the program, but implemented in a way that was beyond prescribed variations).  -Evaluators will recommend sustainment and expansion of adaptations perceived as being effective and beneficial to TSMVs  **Participant Responsiveness:** will be measured by:  -Number of sponsor sessions that TSMVs attend with their sponsor  -Percent of the initial “My Action Plan” completed by TSMVs within 30 days of matching  -Number of monthly updates TSMVs make to their “My Action Plan”  -TSMV satisfaction with the program (1= Strongly Disagree; 5= Strongly Agree)  -“The program has been helpful in my transition to civilian life”  -“I can reach my sponsor when I want to”  -“I would recommend the program to other transitioning Servicemembers”  -“I am satisfied with the program”  **Qualitative data from semi-structured interviews with TSMVs will documents experiences with sponsors and the VSI. |  |
|  |  |  |  |
|  |  |  |  |
| **Maintenance** | “How do I incorporate intervention so that it is delivered over the long term?” | Evaluated after the implementation phase and determined by:  -Number of TSMVs who continue to sign up for the initiative  -Number of sponsors who stay actively engaged in the program  -Number of new sponsors  -Continued VA funding dedicated to VSI sustainment and expansion  **Qualitative data from periodic reflections related to barriers, facilitators, and contextual factors impacting implementation and potential sustainment. |  |
|  |  |  |  |
|  |  |  |  |


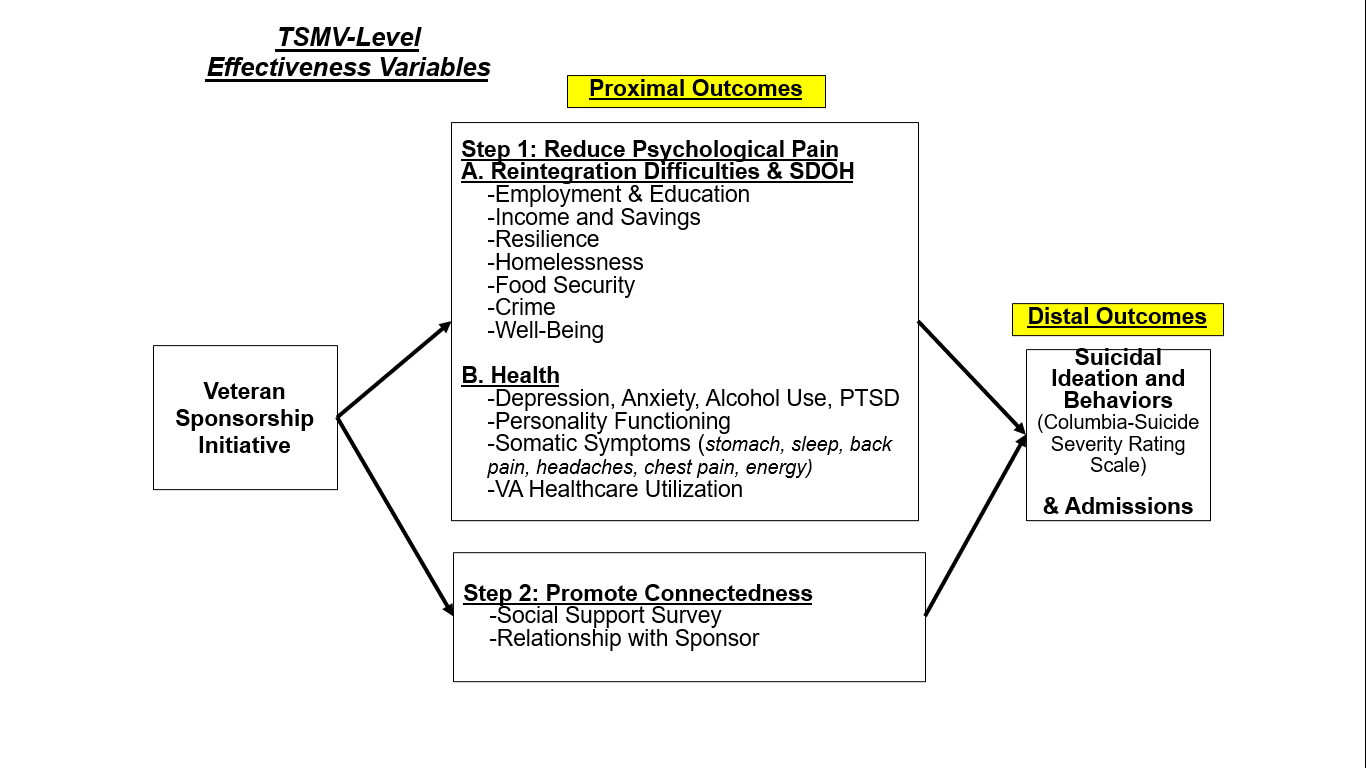


Figure 2. TSMV-level Effectiveness Variables
